# Methane removal and the proportional reductions in surface temperature and ozone

**DOI:** 10.1098/rsta.2021.0104

**Published:** 2021-11-15

**Authors:** S. Abernethy, F. M. O'Connor, C. D. Jones, R. B. Jackson

**Affiliations:** ^1^ Department of Applied Physics, Stanford University, Stanford 94305, USA; ^2^ Department of Earth System Science, Stanford University, Stanford 94305, USA; ^3^ Woods Institute for the Environment and Precourt Institute for Energy, Stanford University, Stanford 94305, USA; ^4^ Met Office Hadley Centre, FitzRoy Road, Exeter EX1 3PB, UK

**Keywords:** methane oxidation, methane-climate responses, methane-ozone responses, negative emissions, methane mitigation

## Abstract

Mitigating climate change requires a diverse portfolio of technologies and approaches, including negative emissions or removal of greenhouse gases. Previous literature focuses primarily on carbon dioxide removal, but methane removal may be an important complement to future efforts. Methane removal has at least two key benefits: reducing temperature more rapidly than carbon dioxide removal and improving air quality by reducing surface ozone concentration. While some removal technologies are being developed, modelling of their impacts is limited. Here, we conduct the first simulations using a methane emissions-driven Earth System Model to quantify the climate and air quality co-benefits of methane removal, including different rates and timings of removal. We define a novel metric, the effective cumulative removal, and use it to show that each effective petagram of methane removed causes a mean global surface temperature reduction of 0.21 ± 0.04°C and a mean global surface ozone reduction of 1.0 ± 0.2 parts per billion. Our results demonstrate the effectiveness of methane removal in delaying warming thresholds and reducing peak temperatures, and also allow for direct comparisons between the impacts of methane and carbon dioxide removal that could guide future research and climate policy.

This article is part of a discussion meeting issue 'Rising methane: is warming feeding warming? (part 1)'.

## 1. Introduction

Atmospheric methane (CH_4_), the second most important greenhouse gas after carbon dioxide (CO_2_), has been steadily increasing at a rate of eight parts per billion (ppb) per year over the past five years [1,2]. Driven primarily by agricultural activities, waste disposal and fossil fuel extraction and use, the global methane concentration is now above 1880 ppb, approximately 2.6 times its preindustrial level [3].

Methane causes substantially more warming than carbon dioxide per unit mass but has a much shorter lifetime [4]. This shorter lifetime is caused primarily by oxidation via tropospheric hydroxyl radicals and, to a lesser extent, oxidation via tropospheric chlorine radicals and microbial consumption in soils [5,6]. Methane emissions induce an atmospheric feedback by decreasing the hydroxyl concentration, thereby increasing methane's lifetime. The strength of this feedback factor, approximately 1.3–1.4, causes the lifetime of a marginal emission, known as the perturbation lifetime, to be significantly higher than the lifetime of methane already in the atmosphere [7,8]. We focus on the perturbation lifetime, approximately 12.4 years, throughout this work. Methane's Global Warming Potential, a ratio of the integrated radiative forcing caused by a pulse emission of a gas relative to that of the same mass of carbon dioxide, is 86 over the first 20 years and 34 over the first 100 years [4]. Accounting for its indirect effects, including increased tropospheric ozone and stratospheric water vapour, methane has contributed nearly half of the present-day effective radiative forcing of carbon dioxide [9–11]. Due to the long-term cumulative impacts of CO_2_, we stress that methane removal should be viewed as a complement to, not a substitute for, carbon dioxide removal and mitigation [12].

Unlike carbon dioxide, methane directly affects air quality through the increased concentration of tropospheric ozone (O_3_) [13–15]. Ozone exposure causes an estimated one million premature deaths annually worldwide due to respiratory illnesses [16]. A 1 ppb reduction in mean global surface ozone is estimated to prevent 50 000 premature deaths per year globally [17]. In addition to preventing premature deaths, reduced ozone levels also improve net primary productivity of vegetation and crop yields, additional benefits of methane removal [13,15].

In this study, we explore the climate and air quality co-benefits and efficacy of removing methane from the atmosphere. Analogous to the numerous studies of carbon dioxide removal [18–21], studies of methane removal are needed to explore its effects on climate, air quality and Earth system feedbacks. The technologies for methane mitigation [22] and removal [23,24] currently being developed require a more detailed understanding of their implications if they are to be implemented at scale.

Our research presents, to our knowledge, the first explicit relationships between methane removal and climate impacts using an emissions-driven atmosphere-ocean coupled Earth System Model (ESM). In previous research into the climate effects of methane removal, some authors have used integrated assessment models, which are faster, but lack the complex detail of general circulation models (GCMs) and ESMs [25]. GCMs and ESMs allow for a more accurate representation of gaseous interactions and have been successfully used to quantify the impacts of methane mitigation in specific scenarios, such as the ozone reduction by 2030 attributable to specific emission control legislation [14,17]. Another study quantified the temperature and ozone reductions attributable to a specific set of seven methane control measures [15]. Yet another study looked at the temperature reductions caused by a 1% or 2% annual decrease in methane concentration using a concentration-driven ESM [26]. Our work builds on these previous studies: we look beyond specific scenarios to extract more general relationships between methane removal and climate impacts through the use of an emissions-driven ESM.

To do so, we use a new capability of the UK Earth System Model (UKESM1) to interactively model the methane cycle, using simulations driven by methane emissions rather than atmospheric concentrations. Methane concentrations are affected by the emissions of multiple gases (including other ozone precursors), so the use of an emissions-driven model allows for direct attribution of the effects of methane emissions on climate, thereby potentially being more useful for policy-makers. Removal is implemented as a negative emission in the same way that carbon dioxide removal has been implemented in similar studies [27]: using shared socioeconomic pathways (SSPs) as baseline scenarios [28–30].

### (a) Removal scenarios

**Figure 1.**
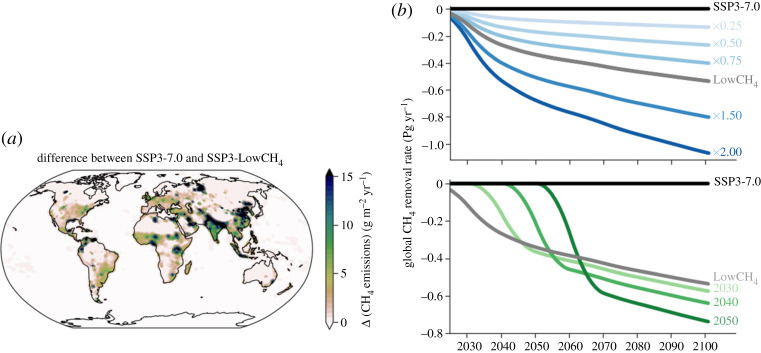
Spatial and temporal removal scenarios for SSP3-7.0. (*a*) The 2020–2100 mean methane emission difference between SSP3-7.0 and SSP3-7.0-LowCH_4_, used as the spatial distribution of removal in all scenarios. Darker colours indicate greater removal. (*b*) Removal scenarios for SSP3-7.0 categorized by amount (blues) and timing (greens). Amount scenarios scale the removal quantity by a multiplicative factor (i.e. ‘x0.25’ is a quarter of the removal of LowCH_4_), while timing scenarios vary the timing of implementation. Data shown are decadal averages. In both panels, mass units are of CH_4_. (Online version in colour.)

We take the methane emissions difference between SSP3-7.0 (‘Regional Rivalry’, a high-emissions future scenario) and SSP3-7.0-LowCH_4_ (the same as SSP3-7.0 other than reduced methane) to create a hypothetical removal scenario that is explicit both spatially (figure 1*a*) and temporally (figure 1*b*). SSP3-7.0-LowCH_4_ was developed based on the methane emissions in SSP3-7.0-LowNTCF [28,31] as an alternative methane-specific pathway for the Aerosol and Chemistry Model Intercomparison Project (AerChemMIP) [29]; its methane emissions are among the lowest of all SSP scenarios. This scale of removal, roughly 40% lower emissions by 2050, is similar to that studied previously by Shindell *et al*. [15], who looked specifically at pollution control measures from 2010 to 2030.

From this removal baseline (LowCH_4_; grey lines in figure 1*b*), we create a set of removal scenarios by varying either the amount removed by multiplying by a constant (0.25, 0.5, 0.75, 1, 1.5 or 2; blue lines in figure 1*b*), or the timing of removal by fixing the cumulative amount removed by 2100 but delaying the start of removal by one or more decades (to 2020, 2030, 2040 or 2050; green lines in figure 1*b*).

These methane removal pathways are then subtracted from the two baseline SSPs used in this work: SSP3-7.0 and SSP1-2.6 (‘Sustainability’, a low-emissions scenario) [31]. Using these contrasting SSPs leads to our modelled scenarios spanning the full range of realistic future methane pathways, from SSP3-7.0 (the highest methane emissions by 2100 of all SSPs [28]) to SSP1-2.6 (one of the lowest methane emissions pathways, from which substantial removal leads to net negative global anthropogenic emissions). The removal scenarios based on SSP3-7.0-LowCH_4_ are associated with future socioeconomic storylines, but they can be applied to other SSPs such as SSP1-2.6 since removal is an additional human activity that is scenario independent. Applying the same removal scenario to both SSPs allows us to isolate methane's impacts and determine if they are affected by the background climate. All ten of the scenarios based on SSP3-7.0 are simulated to 2100, whereas seven of the 10 scenarios based on SSP1-2.6 end before 2100 due to methane concentrations dropping near zero.

### (b) Effective cumulative removal

Cumulative removal is the most common metric for quantifying the climate impacts of carbon dioxide removal, but it is less applicable for methane because of methane's shorter lifetime [32–34]. Removing one unit of methane from the atmosphere today means there is instantaneously one unit less methane, but methane's exponential decay means this amount decreases in the future. Consider, for example, 8.6 years after the removal*—*the half-life of methane, calculated as the 12.4-year lifetime multiplied by ln(2). In 8.6 years, half of the methane that was removed would have oxidized, meaning that the effect of removal would only be half a unit of methane at that time.

We therefore define a new metric—*effective* cumulative removal—to account for the shorter lifetime of methane and allow for a direct comparison with carbon dioxide. Effective cumulative removal is found by integrating the total amount of removed methane that would have otherwise still been in the atmosphere (i.e. that which wouldn't have been oxidized). The effective cumulative removal at time *t* is given by

$$E(t)=\int_{0}^{t}R(t^{\prime }){\text{e}}^{-t^{\prime }/\tau }\text{d}t^{\prime },$$

where *R*(*t′*) is the removal at time *t′* and *τ* is methane's perturbation lifetime. The unit for *E* used here is petagrams (Pg) of methane (where 1 Pg is equivalent to 1 gigaton, 1000 teragrams or 10^12^ kilograms). For scale, the present-day atmospheric burden of methane is approximately 5 Pg, and in SSP3-7.0 it will surpass 8 Pg by 2100. The effective cumulative removal from a ‘pulse’ removal will decay over time, thereby reducing the relative importance of CH_4_ [35]; sustained removal is required for a constant effective cumulative removal. Maintaining a constant effective cumulative removal *E*, for example, requires a constant removal rate equal to *E*/*τ*. This approach is in line with past studies, such as those that use Allen *et al*.'s modified Global Warming Potential GWP*, where equivalences are drawn between the emission rates of short-lived climate forcers and pulse emissions of carbon dioxide [33,36].

### (c) Methane-climate and methane-ozone responses

Emissions-driven simulations allow us to investigate the relationship between methane emissions and climate responses directly, incorporating feedbacks from methane and other ozone precursors on methane lifetime and the climate. Analogous to the transient climate response to cumulative carbon emissions (TCRE), a measure of the net climate response to carbon dioxide emissions, we define a new measure, the methane-climate response (MCR), as

$$\text{ MCR}=\frac{\Delta T}{E}=\left(\frac{\Delta T}{\Delta M}\right)\times \left(\frac{\Delta M}{E}\right),$$

where Δ*T* is the difference in global mean surface temperature, Δ*M* is the mass difference of atmospheric methane and *E* is the effective cumulative removal. Thus, MCR is the product of the temperature response per unit change in atmospheric methane (Δ*T*/Δ*M*) and the atmospheric methane response per unit of effective cumulative removal (Δ*M*/*E*, closely related to the perturbation airborne fraction [20]). MCR measures the sensitivity of the global mean surface temperature to the effective cumulative methane removal; its unit is °C per effective Pg CH_4_ removed.

**Figure 2.**
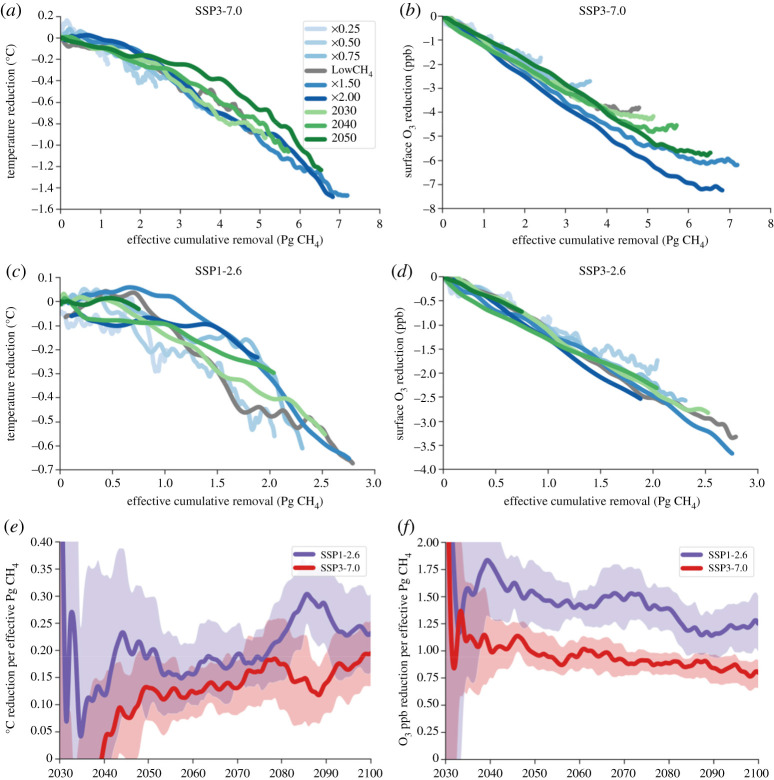
Reductions in surface temperature (left column) and ozone (right column) are proportional to effective cumulative methane removal. Data for all simulations showing effective cumulative removal versus temperature reduction (*a*,*b*) and effective cumulative removal versus ozone reduction (*c*,*d*). (*e*) Methane-climate response over time. (*f*) Methane-ozone response over time. Data in (*e*) and (*f*) are decadal averages and standard deviations that use that SSP as a baseline climate. (Online version in colour.)

Using UKESM1, we find that the modelled relationship between *E* and Δ*M* is sublinear, whereas the relationship between Δ*M* and Δ*T* is superlinear. However, the resulting relationship between *E* and Δ*T* is near-linear, albeit with slight curvature likely due to the delay of the temperature response caused by methane removal (figure 2*a*,*b*). This relationship appears independent of the removal scenario, at least within an SSP. That is to say, within one panel (figure 2*a* or 2*b*) all of the curves follow essentially the same line, illustrating that MCR—the slope of this line—appears to be robust across different removal amounts and timings.

Averaging MCR over all scenarios for each SSP is noisy initially, but more precise estimates are reached by 2080–2100: 0.25 ± 0.07 for SSP1-2.6 and 0.17 ± 0.07 for SSP3-7.0, both in °C per effective Pg CH_4_ removed. Averaging over all simulations for both SSPs, the MCR is 0.21 ± 0.04°C per effective Pg CH_4_ removed. The uncertainties are sufficiently large that the difference is not statistically significant, but the MCR for SSP1-2.6 is slightly higher than for SSP3-7.0 (figure 2*e*), meaning that there is a larger temperature reduction for identical amounts removed. This difference in MCR between SSPs demonstrates how the background climate affects the impacts of methane removal on temperature reduction. Although a plausible explanation, this difference between SSPs is not attributable to differences in the radiative forcing overlap with nitrous oxide between the SSPs. Using simplified equations for radiative forcing [37] and the N_2_O concentrations for both SSPs [31], methane's radiative forcing in SSP3-7.0 would be less than 1% higher if nitrous oxide concentrations were taken from SSP1-2.6. Instead, we attribute (at least partially) the difference between SSPs to their different perturbation lifetimes (and different feedback factors): 9.6 years with a feedback factor of 1.35 for SSP3-7.0 and 8.1 years with a feedback factor of 1.25 for SSP1-2.6.

Since MCR is a new metric, no direct comparisons to published values exist. The closest study to our knowledge is that of Shindell *et al*. [15], who used slightly different emissions modifications to estimate that a 40% reduction in CH_4_ emissions led to a 0.3°C reduction in 2050. Our simulations show that a 40% reduction in CH_4_ by 2050 (namely the difference between an SSP and its LowCH_4_ pathway) leads to a temperature reduction of approximately 0.4°C. Another comparison can be made to the work of Jones *et al*. [26], who used a concentration-driven ESM and found that a 2% compound annual reduction in methane concentration led to a temperature reduction of approximately 0.5°C by 2100. Although not an exact match, our x0.5 scenario follows a similar trajectory to their 2% annual reduction and leads to a reduction of roughly 0.55°C by 2100. This agreement with (and slight revising upward of) literature values demonstrates the utility of MCR, while also illustrating the benefit of its more general definition that can be used across scenarios and ESMs.

Due to the temporal nature of effective cumulative removal, comparisons between methane and carbon dioxide depend on the timescale of interest. The equivalent of MCR for carbon dioxide, the TCRE, is 0.00048 ± 0.0001°C per Pg CO_2_ [38], two orders of magnitude smaller than our MCR estimate of 0.21 ± 0.04°C per effective Pg CH_4_ removed (figure 2). Accounting for the time delay for carbon dioxide removal due to the lagged response of the deep ocean, the TCRE for CO_2_ removal may be even lower [39]. If 1 year of anthropogenic emissions was removed (0.36 Pg CH_4_ [3] and 41.4 Pg CO_2_ [40]), the transient temperature impact would be almost four times larger for methane than for CO_2_ (0.075°C compared to 0.02°C). Using this example, however, maintaining a steady-state response of 0.36 Pg CH_4_ effectively removed would require the ongoing removal of roughly 0.03 Pg CH_4_ yr^−1^, since a removal rate of *E*/*τ* is required to maintain an effective cumulative removal of *E*.

We also define an analogous metric for ozone, the methane-ozone response (MOR), which measures the sensitivity of the global mean surface ozone concentration to effective cumulative methane removal; its unit is O_3_ ppb per effective Pg CH_4_ removed. MOR is a more general and slightly modified version of Fiore *et al*.'s ‘effective emission reduction [14]’; general in that it is defined for any year, and modified in that Fiore *et al*.'s weighting is by the fraction of the steady-state response that has been realized by 2030, whereas the ‘effective’ in our weighting is by the amount of methane that remains out of the atmosphere.

The MOR differs slightly between the extreme climate scenarios of SSP1-2.6 and SSP3-7.0 (figure 2), with estimates by 2080–2100 of 1.2 ± 0.3 for SSP1-2.6, 0.8 ± 0.3 for SSP3-7.0, and 1.0 ± 0.2 for all simulations combined (all in O_3_ ppb per effective Pg CH_4_ removed). One important note is that the near-term benefit of methane removal for surface ozone reduction is even stronger than this estimate would suggest; in figure 2*c*,*d*, the slope is steeper at lower removal levels and then flattens, whereas in figure 2*f*, the ozone reduction is higher at earlier times. The MOR difference between SSPs is likely driven by the significantly higher concentrations of non-methane-ozone precursors in SSP3-7.0, leading to a lower ozone sensitivity to methane [41].

Our estimate of MOR, defined in a novel way based on the integration of removed methane over a period of time, agrees with published values quantifying the effect of CH_4_ on surface ozone concentrations. The ozone reduction (in ppb) due to 20% lower CH_4_ concentration was estimated by two previous multi-model parameterizations to be 0.9 ± 0.14 [42] and 1.05 ± 0.12 [43], while our estimates based on MOR are 1.1 ± 0.2 for SSP1-2.6 and 0.9 ± 0.2 for SSP3-7.0. The global averages we present only capture part of the human health impacts of surface ozone, because outcomes such as premature mortality depend on the spatial distributions of ozone and population. Surface ozone reductions caused by removal from SSP3-7.0 to SSP3-7.0 LowCH_4_, for example, are heavily concentrated in the Northern Hemisphere mid-latitudes (electronic supplementary material, figure S3).

### (d) Delay of warming thresholds in SSP3-7.0

**Figure 3.**
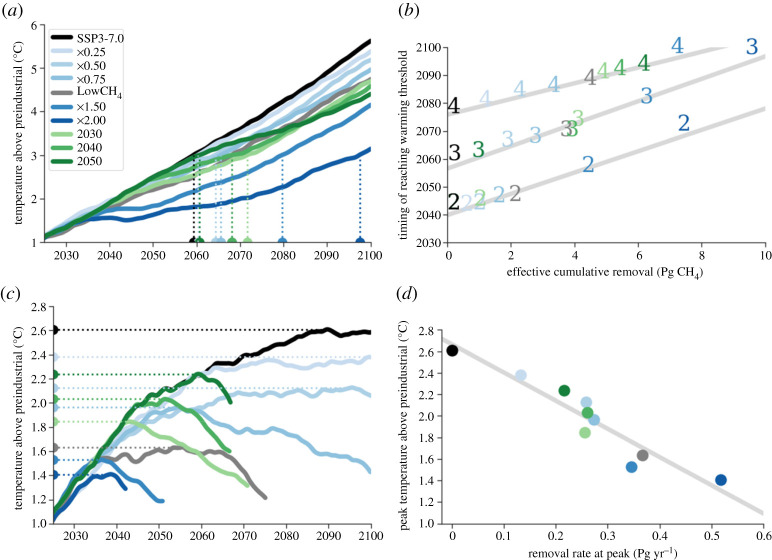
(*a*, *b*) Effective cumulative methane removal causes a linear delay in reaching warming thresholds in SSP3-7.0. (*c*, *d*) Methane removal rate causes a linear reduction in peak temperature in SSP1-2.6. (*a*) SSP3-7.0 decadal average temperatures showing an example of the timing of reaching 3°C warming with dotted lines; (*b*) correlation between effective cumulative removal and timing of warming thresholds with lines of best fit in grey (calculated using linear least-squares regression). Numbers indicate the temperature (in°C) above preindustrial that has been reached; (*c*) decadal average temperatures for SSP1-2.6 scenarios showing peak temperatures with dotted lines; (*d*) correlation between removal rate and temperature at peak along with the best fit line in grey (calculated using linear least-squares regression). (Online version in colour.)

We now examine two specific applications of methane removal, starting with its potential to delay the timing of reaching warming thresholds in SSP3-7.0. In SSP3-7.0, the global mean surface temperature increases near-linearly throughout the twenty-first century, passing the 2, 3 and 4°C warming thresholds above preindustrial temperature by roughly 2040, 2060 and 2080, respectively (figure 3*a*). Methane removal reduces the rate of warming from approximately 0.6°C/decade for SSP3-7.0 down to approximately 0.45°C/decade for SSP3-7.0-LowCH_4_ and approximately 0.25°C/decade for the drastic x2.0 scenario. The timing of reaching the warming thresholds of 2, 3 and 4°C above preindustrial is delayed linearly by effective cumulative methane removal at a rate of 3.8 ± 0.3, 4.0 ± 0.2 and 2.8 ± 0.2 years, respectively, per effective Pg CH_4_ removed (figure 3*b*). We note that this is for SSP3-7.0, an extremely emissions-heavy scenario, and using UKESM1, a model with a higher-than-average climate sensitivity [44]. We therefore hypothesize that our estimated delays are likely a lower bound since we expect longer delays for scenarios with a more gradual temperature increase or models with lower climate sensitivity.

### (e) Reduction of peak temperature in SSP1-2.6

Our second application is the potential for methane removal to reduce the severity of the temperature peak that occurs in SSP1-2.6. In previous research, methane reduction has been shown to increase the available carbon dioxide budget, meaning that more carbon dioxide can be emitted while still staying below a certain temperature threshold [13]. We instead consider carbon dioxide concentrations that are specified by the SSPs [31] to isolate the relationship between methane removal and the reduction of the peak temperature, *T*(*t*_peak_). The peak temperature is reduced from 2.6°C above preindustrial in SSP1-2.6 to 1.6°C in the LowCH_4_ scenario and 1.4°C in the x2.0 scenario (figure 3*c*). There is a linear relationship between the peak temperature and the methane removal rate at the timing of the peak (figure 3*d*):

$$\Delta T(t_{\text{peak}})=cR(t_{\text{peak}}),$$

where *R*(*t*_peak_) is the removal rate at the time of peak temperature. The slope, *c*, a measure of the responsiveness of the peak temperature to removal rate, is −2.6 ± 0.6°C per Pg CH_4_ yr^−1^.

We find that the methane removal rate is the best predictor of the peak temperature in SSP1-2.6. This finding agrees with previous research showing that the peak temperature in optimistic scenarios such as SSP1-2.6 is best predicted by a linear combination of the cumulative carbon dioxide emissions and the instantaneous emission rate of methane at the time of the peak [32,36,45,46].

## 2. Discussion

One important value used in the calculation of effective cumulative removal is *τ*, the methane perturbation lifetime. We presented our results using the average modelled perturbation lifetime for each SSP from 2020–2100, 9.6 years for SSP3-7.0 and 8.1 years for SSP1-2.6. These lifetimes incorporate tropospheric and stratospheric oxidation and soil sinks as well as the feedback factor that methane has on its own lifetime, which was calculated to be 1.35 for SSP3-7.0 and 1.25 for SSP1-2.6, in good agreement with literature values such as 1.28 for UKESM1 [7,9]. Using the modelled perturbation lifetime, instead of simply using the average ESM value of 12.4 years, has a significant impact on MCR and MOR. If we had used 12.4 years instead of the UKESM1 modelled lifetime, values for MCR and MOR would be reduced by 20–30% (electronic supplementary material, table S1).

To reduce any potential biases that are present in UKESM1 and increase the robustness of our results, multi-model analyses of MCR and MOR should be undertaken. This would be best done with a standardized research agenda and a Methane Removal Model Intercomparison Project, similar to what has been done for carbon dioxide [19,27,47], potentially with model weighting based on observational constraints [48]. To that end, we encourage the development and refinement of interactive-methane emissions-driven configurations in other ESMs.

We analysed surface temperature and ozone concentration in this work because they are important metrics for climate and human health, but there are additional aspects to consider when calculating the full impacts of methane removal. One consideration is the potential unintended atmospheric chemistry effects of methane removal. For example, removal technologies that oxidize methane to carbon dioxide may inadvertently oxidize partially to carbon monoxide (CO) or methanol (CH_3_OH) [24]. Furthermore, removal technologies must be compared in terms of costs, energy, land and water usage, and social implications of implementation. Our results show that methane removal could play a critical role in improving air quality and reducing temperatures, thereby potentially complementing the negative emissions of carbon dioxide required to meet the Paris climate goals.

## 3. Methods

The novel modelling capability that allows a quantification of climate impacts directly from methane emission reductions is the new ‘emissions-driven’ configuration based on version 1.0 of the UK's ESM, UKESM1 [49]. Instead of following the ‘concentration-driven’ models used in past work such as the Coupled Model Intercomparison Project Phase 6 (CMIP6), where the concentration at each timestep is specified, emissions-driven models crucially account for feedbacks in the methane cycle by incorporating interactive methane sources and sinks.

The experimental set-up used here is based on UKESM1 [49], a state-of-the-art coupled ESM. It includes the United Kingdom Chemistry and Aerosol (UKCA) model [50,51] to represent troposphere-stratosphere gas-phase [52] and aerosol-phase [53] composition, the Joint UK Land Environment Simulator (JULES) model [54–57] to simulate terrestrial biogeochemistry and dynamic vegetation, and the Model of Ecosystem Dynamics, nutrient Utilisation, Sequestration and Acidification (MEDUSA [58]) for dynamic ocean biogeochemistry. The model resolution is N96L85-ORCA1; this equates to an atmospheric resolution of 1.25° × 1.875° in the horizontal, with 85 levels in the vertical from the surface up to the model top at 85 km. The ocean horizontal resolution is 1°.

In the default ‘concentration-driven’ configuration of UKESM1, the global mean methane concentration is prescribed as a lower boundary condition in UKCA [52] and follows specified concentrations based on historical observations [59] or SSPs [31]. Methane concentrations above the surface are calculated interactively. Although UKESM1 includes methane wetland emissions, the largest natural source of methane that is subject to a large feedback [60], they are diagnostic only and methane soil uptake is not considered. Anthropogenic and biomass burning emissions of non-methane-ozone precursors for the historical period are prescribed using Hoesly *et al*. [61] and van Marle *et al*. [62], respectively, and for the future SSPs using Gidden *et al*. [28] . Lightning emissions of nitrogen oxides and biogenic emissions of volatile organic compounds are interactive [49,52], while all other natural emissions of non-methane-ozone precursors are prescribed (details of which can be found in Archibald *et al*. [52] and Sellar *et al*. [49]).

In the ‘emissions-driven’ configuration used here, by contrast, the prescribed global mean surface methane concentration is replaced with emission sources [28,61,62]. Methane surface removal is treated explicitly, and the interactive wetland emissions from the JULES land surface model [63,64] are coupled to UKCA. Other natural methane emission sources are prescribed. Further details on UKESM1 and its ‘emissions-driven’ configuration can be found in Sellar *et al*. [49] and Folberth *et al*. (GA Folberth, CD Jones, FM O'Connor, N Gedney, AA Sella, A Wiltshire 2021, submitted), respectively.

## Data accessibility

The data required to replicate and build on the results of this paper can be found on the CEDA Archive at https://catalogue.ceda.ac.uk/uuid/1bd597257788460189e62f46d60e0b0e. Raw three-dimensional data output from UKESM1 is available from the corresponding author on reasonable request.

## Code accessibility

We are unable to provide source code for UKESM1 due to intellectual property rights restrictions. The Met Office Unified Model (MetUM) is available for use under licence. A number of research organizations and national meteorological services use the UM in collaboration with the Met Office to undertake basic atmospheric process research, produce forecasts, develop the UM code, and build and evaluate ESMs. For further information on how to apply for a license, see http://www.metoffice.gov.uk/research/modelling-systems/unified-model (last access: 14 August 2019). UM and JULES simulations are compiled and run in suites developed using the Rose suite engine (http://metomi.github.io/rose/doc/html/index.html, last access: 14 August 2019) and scheduled using the Cylc workflow engine (https://cylc.github.io/cylc/, last access: 14 August 2019). Both Rose and Cylc are available under version 3 of the GNU General Public License. In this framework, the suite contains the information required to extract and build the code as well as configure and run the simulations. Each suite is labelled with a unique identifier and is held in the same revision-controlled repository service in which we hold and develop the model's code. This means that these suites are available to any licensed user of both the UM and JULES. Python code for figure generation is available from the corresponding author upon request.
